# A Comparative Analysis of the Camera-like Eyes of Jumping Spiders and Humans

**DOI:** 10.3390/vision6010002

**Published:** 2021-12-31

**Authors:** Irina P. Shepeleva

**Affiliations:** Laboratory of Visual Physiology, Pavlov Institute of Physiology, Russian Academy of Sciences, Makarova emb. 6, 199034 St. Petersburg, Russia; i.p.shepeleva@yandex.ru

**Keywords:** camera-like eye, fovea, jumping spiders, humans

## Abstract

Among invertebrates, jumping spiders are one of the few groups whose representatives have camera-like eyes, and the only group whose representatives have fovea. The latter is present in the camera-like eyes of representatives of some groups of vertebrates, including humans. Based on the literature data, a comparative analysis of the camera-like eyes of jumping spiders and humans was carried out, in the course of which the similarities and differences in the properties and functions of their basic components were identified. The presented data are necessary for the formation of knowledge about jumping spiders as model animals for studying the functioning of the visual system.

## 1. Introduction

All vertebrates, including humans, and some invertebrates have camera-like eyes. However, not all vertebrates have fovea in their eyes, as in humans. Among invertebrates, it is found only in jumping spiders [1,2,3,4,5,6,7]. Jumping spiders represent the largest family of spiders, which has more than 6000 species and whose size usually varies from 1 to 25 mm [8,9]. Jumping spiders, with the exception of one herbivorous species, *Bagheera kiplingi* Peckham and Peckham, 1896 [10], are predators who hunt by the help of the vision both in high and low brightness conditions [11,12].

As a rule, jumping spiders have four pairs of camera-like eyes of two species: one pair of principle eyes—antero-median and three pairs of secondary eyes—antero-lateral, postero-median and postero-lateral. Each pair of eyes, in addition to features in location, shape, size and structure, has such individual characteristics as the value of the spatial resolving power, color perception, size of the field of view, light perception and the ability to perceive the depth of space. All of them are necessary for jumping spiders to perform different visual tasks, but it is impossible to combine them in one pair of eyes, for example, as in humans, due to their small size [13,14]. The principle and secondary eyes of jumping spiders have characteristics similar to those of the fovea—the zone of the central region of the retina and the peripheral region of the retina of the human eye, respectively.

The principle eyes of jumping spiders are characterized by a high value of the spatial resolving power, color perception, a narrow field of view, a low value of the sensitivity to light and the ability to perceive the depth of space in the same way as the fovea of the human eye, where cones are mainly located [1,2,13,15,16,17]. The highest value of the spatial resolving power (Nyquist frequency) of the principle eyes of jumping spiders, such as *Portia fimbriata* Doleschell, 1859, is 12.5 cycles/degree and has no analogues among animals with eyes of comparable size [18] (the parameter was calculated by the author based on data from [18]). It is only five times lower than the maximum value of the spatial resolving power of the fovea of the human eye, which is 60 cycles/degree [19,20]. In most species of jumping spiders, photoreceptor cells in the retina of the principle eyes provide dichromatic vision in the ultraviolet (λ_max_ ≈ 377 nm) and green (λ_max_ ≈ 530 nm) parts of the electromagnetic radiation spectrum, as in *Menemerus confusus* Koch, 1878 [21], whereas in some species trichromatic vision is additionally in the red (λ_max_ ≈ 626 nm) part of the spectrum, as in *Habronattus pyrrithrix* Chamberlin, 1924 [22], and tetrachromatic vision is additionally in the blue (λ_max_ ≈ 480–500 nm) part of the spectrum, as in *Maratus* sp. Karsch, 1878 [23]. In humans, cones are normally the basis of trichromatic vision in the violet-blue (λ_max_ ≈ 430 nm), yellow-green (λ_max_ ≈ 530 nm) and yellow-red (λ_max_ ≈ 560 nm) parts of the spectrum [24,25]. Depending on the species of jumping spider, the size of the field of view of the fovea, which is elongated in the vertical direction, is estimated from 0.8°, as in *P. fimbriata* [26], to 5.0°, as in *Metaphiddipus aeneolus* Curtis [17], horizontally and 20°, as in *M. aeneolus* [17], vertically. It is, respectively, 6.3–1.0 times smaller and 4.0 times larger than the size of the human eye fovea visual field, which is estimated at 5.0° [27]. The principle eyes of jumping spiders and fovea of the human eyes, in contrast to the high spatial resolving power, have low sensitivity to light, which makes it possible to implement the functions of central (form) vision and color perception in conditions of high light brightness [13,15,16]. Jumping spiders, for example, *Hasarius adansoni* Audouin, 1826, use their principle eyes to perceive the depth of space due to a unique monocular cue for animals—defocusing images of observed objects on the retina [28]. In jumping spiders in the principle eyes, the light-sensitive parts of photoreceptor cells form four layers, two of which—the deepest first and second layers—are sensitive to green light [1]. The perception of the depth of space is achieved by comparing the defocused image obtained by the second layer with the focused image obtained by the first layer [28]. Humans perceive the depth of space mainly due to a binocular cue—binocular disparity, which underlies binocular stereoscopic vision [16,29,30].

In comparison with the principle eyes, the secondary eyes of jumping spiders are characterized by a low value of the spatial resolving power, color perception (only postero-median eyes), a wide field of view, a high value of the sensitivity to light and the ability to perceive the depth of space (only antero-lateral eyes) as well as the peripheral region of the retina of the human eye, where the rods are mainly located [1,2,13,15,16,17]. The value of the spatial resolving power of the three species of secondary eyes of jumping spiders differs and varies depending on the species of spider: in antero-lateral eyes—from 0.3 cycles/degree, as in *M. aeneolus* [1], to 1.3 cycles/degree, as in *Evarcha blancardi* Scopoli, 1763 [31], in postero-lateral eyes—from 0.3 cycles/degree, as in *M. aeneolus* [1], to 0.6 cycles/degree, as in *Epiblemum* sp. Hentz, 1832 [31], and in postero-median eyes is 0.05 cycles/degree, as in *Epiblemum* sp. [31] (all parameters were calculated by the author based on data from [1] and [31] using the formula from [2]). It is 125–29, 125–62 and 748 times lower, respectively, than the value of the spatial resolving power of the peripheral region (far periphery) of the human retina, which is 37.4 cycles/degree (the parameter was calculated by the author based on data from [7]). In jumping spiders, photoreceptor cells in the retina of antero-lateral and postero-lateral eyes provide monochromatic vision in the green (λ_max_ ≈ 535–540 nm) part of the spectrum, as in *M. confusus* [21], postero-median eyes—dichromatic vision in the ultraviolet (λ_max_ ≈ 370 nm) and blue (λ_max_ ≈ 480 nm) parts of the spectrum, as in *H. adansoni* [32]. In humans, rods determine monochromatic vision in the green (λ_max_ ≈ 510 nm) part of the spectrum [24]. The size of the field of view of three pairs of secondary eyes of jumping spiders, for example, *Trite planiceps* Simon, 1899, is estimated to be approximately 330° horizontally [33]. It is almost twice the size of the field of view of one pair of human eyes, which is estimated to be approximately 180° horizontally [34]. The secondary eyes of jumping spiders and the peripheral region of the retina of the human eye, in contrast to the low spatial resolving power, have a high sensitivity to the light, which makes it possible for orientation in space under conditions of low light brightness [12,13,15,16]. Jumping spiders with the help of secondary antero-lateral eyes and humans perceive the depth of space thanks to binocular stereoscopic vision [16,30,33].

Thus, with the help of secondary eyes, jumping spiders determine the presence of a moving object in their field of view at a distance of up to 3 m and initiate the movement of the body and the retinas of the principle eyes in order to place an object on them for its assessment. With the help of the principle eyes, jumping spiders perceive the form, details and colors of objects, which ensures their recognition at a distance of up to 30 cm, and also assess the size of objects and the distance to them [1,11,13,17,28,35]. In addition, in these invertebrates, for example, in *Servaea vestita* Koch, 1879, as in humans, hyperacuity of vision was detected—the ability to determine the angular displacement of an object, which is significantly less than the angular distance between the centers of neighboring photoreceptor cells [36]. The purpose of the work is to compare the camera-like eyes of jumping spiders and humans. The human eye was chosen as a typical example of the camera-like eye of vertebrates. Comparative analysis is necessary to create an idea of jumping spiders as model animals for studying the functioning of the visual system.

## 2. General Characteristics of the Camera-like Eyes of Jumping Spiders and Humans

**Figure 1:**
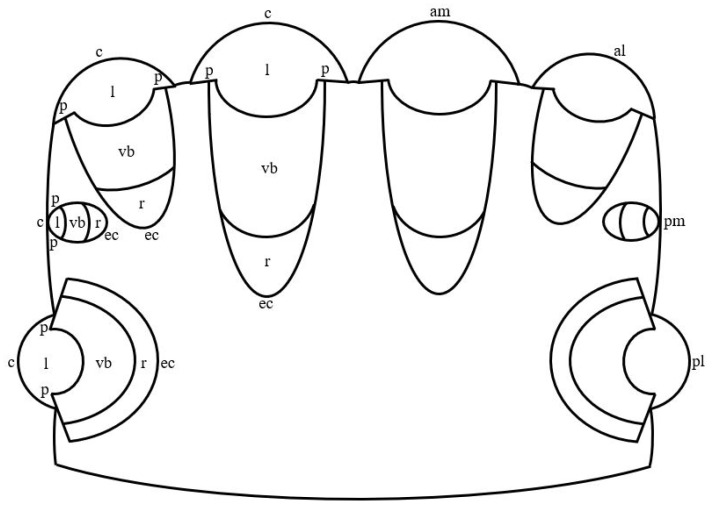
Schematic drawing of the camera-like eyes of jumping spiders. am: antero-median eyes; al: antero-lateral eyes; pm: postero-median eyes; pl: postero-lateral eyes; c: cornea; l: lens; p: pupil; vb: vitreous body; r: retina; ec; eye capsule.

**Figure 2:**
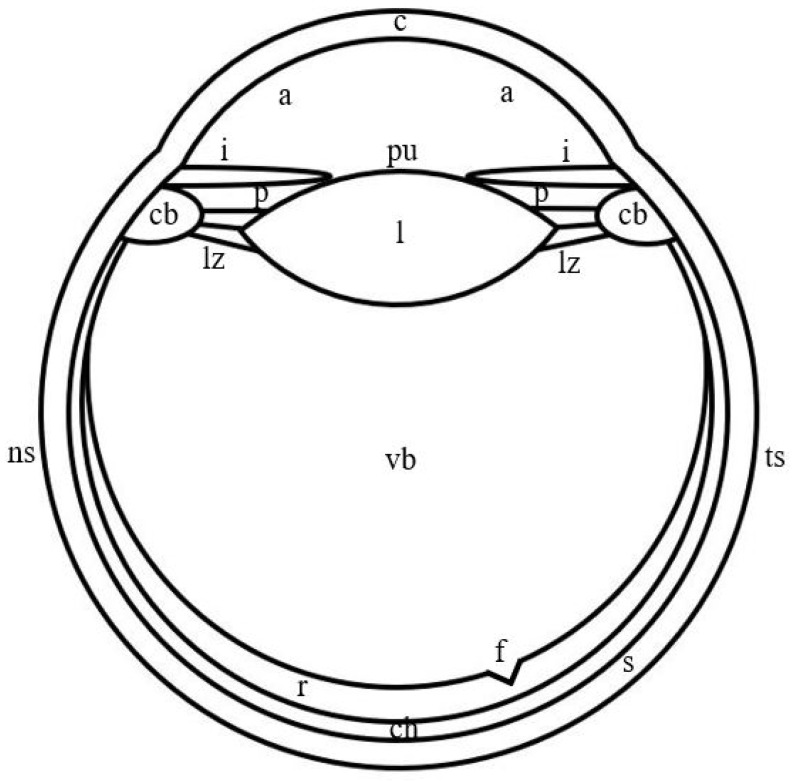
Schematic drawing of the camera-like eye of humans. c: cornea; a: anterior chamber; i: iris; pu: pupil; p: posterior chamber; l: lens; lz: ligament of Zinn; cb: ciliary body; vb: vitreous body; f: fovea; r: retina; ch: choroid; s: sclera; ns: nasal side; ts: temporal side.

In jumping spiders, four pairs of eyes are located on the cephalothorax in three rows (Figure 1). The first row is represented by the principle antero-median and secondary antero-lateral eyes, the second row—by secondary postero-median eyes, which in more developed groups, for example, in *Lagnus* sp. Koch, 1879 and *Plexippus validus* Urquhart, 1893 [37], can be rudimentary, and the third row—by secondary postero-lateral eyes. The antero-median and antero-lateral eyes look forward, the rest of the eyes—to the sides [13]. In humans, one pair of eyes is located in the front of the head in the orbits and is directed forward [15]. In jumping spiders, the antero-median, antero-lateral and postero-median eyes have a tubular shape, whereas the shape of the postero-lateral eyes is close to spherical [1,33,38]. Three shapes of eyes have been described in humans: spherical; oblate ellipsoid; prolate ellipsoid [39]. In jumping spiders, for example, in *M. aeneolus* [1] and *Metaphiddipus harfordi* Peckham and Peckham, 1896 [40], the largest are the antero-median eyes (about 800 microns along the anterior-posterior axis), the smaller and comparable to each other are the antero-lateral (300–500 microns) and postero-lateral (200–300 microns) eyes, and the smallest are the postero-median eyes (100 microns). In humans, the average length of the eye along the antero-posterior axis reaches 22.0–24.8 mm [41]. The eyes of jumping spiders consist of five components: the outer shell, represented by the cornea and the eye capsule; the inner shell, represented by the retina; the pupil; the lens; the vitreous body (Figure 1) [13,42]. The human eye consists of eight components: the outer shell, represented by the cornea and sclera; the anterior chamber, filled with aqueous humor; the middle shell, represented by the choroid, ciliary body and iris; the pupil; the posterior chamber, filled with aqueous humor; the inner shell, represented by the retina; the lens; the vitreous body (Figure 2) [15]. Thus, the camera-like eyes of jumping spiders and humans differ significantly in size (27.5–248 times) and structure, but at the same time contain five identical components: the outer shell; the inner shell; the pupil; the lens; the vitreous body. Further, in the work of jumping spiders and humans, the properties and functions of these eye components are analyzed.

## 3. The Outer Shell of the Camera-like Eyes of Jumping Spiders and Humans

The outer shell of the eyes in jumping spiders is represented by the cornea and the eye capsule, in humans—by the cornea and sclera [15,43].

### 3.1. Cornea

The cornea of jumping spiders and humans is the anterior smaller part of the outer shell and the lens of the eyes (Figure 1 and Figure 2). It is characterized by transparency and colorlessness [15,43,44]. In jumping spiders, the cornea is formed by the cuticle of the carapace, therefore it is a layered structure. The cuticle is of secretory origin and mainly consists of polysaccharide chitin immersed in a protein matrix [43,45]. In humans, the cornea is formed by epithelial and connective tissue. In the direction from the outside to the inside of the eye, six layers are distinguished in it: a layer formed by a multilayer squamous non-keratinizing epithelium; anterior limiting membrane; stroma—a layer of dense fibrous regular connective tissue, which consists of an extracellular matrix represented by collagen and elastic fibers in the ground substance, and several types of cells, the main of which are keratocytes; Dua’s layer—a thin high-strength layer of collagen; posterior limiting membrane; a layer formed by a single-layer single-row squamous epithelium [15,44,46,47]. In jumping spiders and humans, the outer and inner surfaces of the cornea form its anterior and posterior refractive surfaces, respectively. Both refractive surfaces have a hemispherical shape in jumping spiders and an aspherical shape in humans [1,27]. Judging by the photographs of the sections of the eyes of jumping spiders, for example, *M. harfordi* [40] и *Phiale magnifica* Banks, 1909 [38], their cornea, similar to the human cornea [15], belongs to the type of thin convex-concave, or meniscus, lenses. The refractive index of the cornea in jumping spiders is taken equal to 1.55, in humans it is 1.376 [27,40].

In jumping spiders and humans, the cornea performs several identical functions: shaping, supporting, protective, light-refracting and light-guiding [2,15]. The cornea of jumping spiders completely absorbs ultraviolet radiation with wavelengths shorter than 290 nm and, along with other invertebrates and vertebrates, is most likely capable of absorbing infrared radiation [48,49]. The human cornea reduces the spherical aberration of the eye, and also completely absorbs ultraviolet radiation with wavelengths shorter than 300 nm and infrared radiation with wavelengths longer than 2500 nm [27,50].

Thus, the cornea of jumping spiders and humans has more of the same properties and performs more of the same functions (Table 1).

### 3.2. Eye Capsule and Sclera

The eye capsule in jumping spiders and the sclera in humans is the posterior larger part of the outer shell of the eyes (Figure 1 and Figure 2). In jumping spiders, the eye capsule is a connective tissue shell, the properties and structure of which are not described in the literature [51]. However, the nature of the staining of the eye capsule in photographs of semithin sections of the eyes, for example, *Plexippus validus* Urquhart, 1893 [52,53], *Synemosyna americana* Peckham and Peckham, 1885 [38] and *P. fimbriata* [54], for review see [55], indicates its transparency and colorlessness. In humans, the sclera is an opaque white shell, which is formed by connective tissue and in which three layers are distinguished from the outside to the inside of the eye: episclera—a layer of loose fibrous irregular connective tissue; stroma—a layer of dense fibrous irregular connective tissue; a brown plate—a layer of loose fibrous irregular connective tissue. All layers consist of the extracellular matrix, represented by collagen and elastic fibers in the ground substance, and several types of cells, the main of which are sclerocytes. The layers of the sclera differ in the diameter of collagen fibers, the density of arrangement and spatial orientation of elastic fibers and may differ in the density of arrangement and spatial orientation of collagen fibers, as well as in cellular composition [15,44,56,57]. The refractive index of the eye capsule in jumping spiders is unknown. In humans, the refractive index of the sclera is 1.41 [58]. Since both shells are connective tissue, it can be assumed that the values of their refractive indices are similar.

In jumping spiders and humans, the eye capsule and sclera perform several identical functions: shaping, supporting and protective. In jumping spiders, the eye capsule serves as a site of attachment of the muscles that drive the retina. In humans, the sclera is the site of attachment of the oculomotor muscles and the ciliary muscle and is involved in the outflow of intraocular fluid [15,43,44].

Thus, the eye capsule of jumping spiders and the human sclera has more of the same properties and performs more of the same functions (Table 1).

## 4. The Inner Shell of the Camera-like Eyes of Jumping Spiders and Humans

The inner shell of the eyes in jumping spiders and humans is represented by the retina, which is its only section. In jumping spiders, the retina of the antero-median eyes can perform the same movements as the human eyes: three pairs of muscles ensure its movement up and down, left and right, and rotation around its axis [17,27]. In jumping spiders, the retina of the antero-median eyes is V-shaped [1]. Photographs of eye sections permit the determination of the shape of the retina of antero-lateral and postero-median eyes as U-shaped, as in *T. planiceps* [33] and *M. harfordi* [40], respectively; the shape of the retina of postero-lateral eyes is close to the shape of an open sphere, as in *T. planiceps* [33] и *P. magnifica* [38] (Figure 1). The human retina has one shape or another depending on the shape of the eye, namely: an open sphere; an open oblate ellipsoid; an open prolate ellipsoid (Figure 2) [39]. In the retina of the listed species of jumping spiders and humans, two parts can be distinguished: optic and non-optic. In jumping spiders, the optic part in the antero-median and antero-lateral eyes occupies only the base of the retina, in the postero-lateral and postero-median eyes—the base and partially the lateral walls of the retina. The optic part in the antero-median eyes has the shape of a boomerang, in the antero-lateral, postero-lateral and postero-median eyes—a hemispherical shape. The non-optic part in all species of eyes is the remaining part of the retina to the pupil [1,33,38,40]. In humans, the optic part occupies the base and completely the lateral walls of the retina and has a shape corresponding to the shape of the retina, namely: an open sphere; an open oblate ellipsoid; an open prolate ellipsoid. The non-optic part is the region of the ciliary body and the iris [15,59]. In jumping spiders in the antero-median eyes, the optic part consists entirely of the fovea, in which the central and peripheral regions can be distinguished. There is no fovea in antero-lateral, postero-lateral, and postero-median eyes [1,33,38,40]. In humans, the optic part is also divided into central and peripheral regions, which in turn are subdivided into zones. The central region of the retina (macula) contains the fovea (central pit), in which the foveola (bottom) with the center (umbo), the declivity and the thick margin are distinguished, and which is surrounded by the parafovea and perifovea. In the peripheral region, with the distance from the central region, the near, middle, far and extreme periphery are distinguished [59]. In jumping spiders, the fovea varies and at the same time differs from the human fovea in shape, curvature, depth and width relative to the retina. Judging by the photographs of the sections of the eyes of jumping spiders, in light-loving species, such as *P. magnifica* and *Jollas geniculatus* Simon, 1901 [38], compared with shade-loving species, such as *Itata completa* Banks, 1929 and *Fluda princeps* Banks, 1929 [38], the fovea is more conical, curved, deep and narrow. In these species of jumping spiders, compared with humans, the fovea looks less rounded and curved, smaller and wider. In jumping spiders, the fovea lies on the optical axis of the eye, is colorless and contains all the layers of the retina in the central region, whereas in humans it is slightly displaced temporal and inferior from the optical axis, is colored yellow and lacks some of the layers [33,38,59,60,61].

In jumping spiders, the retina is non-inverted in the antero-median eyes, inverted in antero-lateral, postero-lateral and postero-medial eyes. The optic part of the retina of the principle and secondary eyes is formed by epithelial and nervous tissue. There is no data in the literature on how many layers and which layers are distinguished in the optic part of the retina. It is known that in the principle eyes, the optic part consists of two types of cells—photoreceptor and pigmented supporting cells, in the secondary eyes—of three types of cells—photoreceptor, pigmented supporting and non-pigmented supporting, or glial cells. The non-optic part of the retina of the principle and secondary eyes is formed by epithelial tissue. Just as for the optic, for a non-optic part, there is no data on how many layers and which layers are distinguished in it in the literature. In the principle and secondary eyes, the non-optic part consists of one type of cells—pigmented supporting cells [1,13,33,37,38,40].

The human retina is inverted. The optic part of the retina is formed by epithelial and nervous tissue. With the exception of the fovea, 10 layers are distinguished from the outside to the inside of the eye: 1—a layer of a single-layer single-row cubic pigment epithelium; 2—a layer of rods and cones, represented by the outer and inner segments of these cells; 3—an outer limiting membrane formed by the processes of glial cells; 4—the outer nuclear layer, in which the nucleated parts of rods and cones are located; 5—the outer plexiform layer—the zone of synaptic contacts between photoreceptor, bipolar and horizontal cells; 6—the inner nuclear layer, in which the nucleated parts of horizontal, bipolar, amacrine, interplexiform and glial cells are located; 7—the inner plexiform layer—a zone of synaptic contacts between bipolar, amacrine and ganglion cells; 8—a layer of ganglion cells; 9—a layer of nerve fibers, consisting of axons of ganglion cells; 10—an inner limiting membrane formed by basal membranes and processes of glial cells. In this case, 10 layers consist of eight types of cells—pigmented, photoreceptor, horizontal, bipolar, amacrine, interplexiform, ganglion and glial. Five layers remain in the fovea: the first–fourth and the tenth. The five layers consist of three types of cells—pigmented, photoreceptor and glial. The non-optic part of the retina is formed by epithelial tissue. One layer is distinguished in it: a layer of a single-layer single-row cubic pigment epithelium. The non-optic part consists of one type of cells—pigmented cells [15,27,62]. Between the first and third layers of the optic part of the retina there is a subretinal space, which is filled with an extracellular matrix called the interphotoreceptor. The space between other types of cells is filled with an extracellular matrix called the retinal. The extracellular matrix of the retina consists of organic and inorganic compounds [63,64].

In jumping spiders, the refractive index of the retina is 1.369, in humans it is approximately 1.360 [55,65].

In the principle and secondary eyes of jumping spiders and human eyes, the retina performs several identical functions: shaping, supporting and light-guiding (some layers of the retina). The retina of the principle eyes of jumping spiders and the fovea of the human eye provide the realization of two identical functions: central (form) vision, which is characterized by high spatial resolving power, and color perception. The retina of the secondary eyes of jumping spiders and the peripheral region of the human retina also provide the realization of two identical functions: peripheral vision, which is characterized by a wide field of vision, and light perception. It should be noted that in jumping spiders, the retina of one of the three species of secondary eyes—postero-median eyes—is able to perceive colors [1,13,15,17,66]. The retina of the principle and secondary antero-lateral eyes and human eyes participates in the implementation of the function of perception of the depth of space: by the help of the principle eyes, jumping spiders perceive the depth of space due to a monocular cue—defocusing images of objects on the retina; by the help of the secondary antero-lateral eyes, jumping spiders perceive the depth of space due to binocular stereoscopic vision in the same way as humans [16,28,67]. In addition, in humans, the central region of the retina of the eye reduces chromatic aberration by attenuating radiation in the blue part of the spectrum [68,69].

Thus, the retina of jumping spiders and humans has more different properties and performs more of the same functions (Table 1).

## 5. The Pupil of the Camera-like Eyes of Jumping Spiders and Humans

Depending on the light conditions of the habitats of jumping spiders, the pupil in their eyes is absent or expressed to varying degrees [42]. So, shade-loving species, such as *I. completa*, do not have a pupil, in contrast with light-loving species, such as *P. magnifica* [38]. In the latter, the pupil can reduce the flow of light entering the eye by up to 50% [42]. Judging by the photographs of eye sections, in the principle and secondary eyes of jumping spiders, for example, *T. planiceps* [33] and *P. magnifica* [38], the pupil is located at some distance from the cornea in the same way as in human eyes [15]. In these species of jumping spiders, the pupil is approximately at the level of the middle of the lens [33,38], in humans—in front of the lens [15]. In the listed species of jumping spiders, the pupil is an opening in the non-optic part of the retina, which is the only part of the inner shall of the eyes [33,38]. As mentioned above, in the principle and secondary eyes, the non-optic part of the retina is formed by pigmented cells. The pupil has a round shape and a constant diameter [42]. In humans, the pupil is an opening in the iris, which is one of the three sections of the middle shell of the eye. The iris is formed by epithelial, connective and muscular tissue. In the direction from the outside to the inside of the eye, three layers are distinguished in it: a layer formed by a single-layer single-row squamous epithelium; stroma—a layer of loose fibrous irregular connective tissue, which consists of an extracellular matrix represented by collagen fibers in the ground substance, and several types of cells, the main of which are fibroblasts and melanocytes, and also contains muscles that constrict and dilate the pupil; a layer formed by a two-layer cubic pigment epithelium. The number and location of melanocytes in the iris stroma determine its color [70,71,72]. The shape of the pupil varies depending on the lighting conditions: in the light it is more rounded with a long axis almost horizontally, and in the dark it is more ellipsoid with a long axis almost vertically [73]. The pupil diameter also reflexively changes: from 1 mm in bright light to 8 mm in the dark [27,72,74]. The center of the pupil is located slightly (0.5 mm) nasal and superior relative to the optical axis of the eye and can shift when the pupil diameter changes [27,75,76].

In jumping spiders and humans, the pupil provides light penetration into the eye [15,42]. In humans, the pupil regulates the amount of light reaching the retina [27]. Changing the pupil size acts as a buffer during the transition from bright light to dark [77]. The size of the pupil affects the size of the blue circle with refractive errors, spherical and chromatic aberration and diffraction, and, consequently, the degree of their expression, as well as the depth of field and focus of the eye. The pupil acts as a channel for aqueous humor between the anterior and posterior chambers of the eye and prevents an increase in pressure in the posterior chamber [72,77].

Thus, the pupil of jumping spiders and humans has more different properties and performs more different functions (Table 1).

## 6. The Lens of the Camera-like Eyes of Jumping Spiders and Humans

In jumping spiders, the lens adheres to the cornea and, judging by photographs of eye sections, for example, *M. harfordi* [40] and *T. planiceps* [33], occupies an insignificant part of the eye cavity of all species, whereas in humans it is located at a distance from the cornea and occupies an even more insignificant part of the eye cavity (Figure 1 and Figure 2) [15]. The lens of all species of eyes of jumping spiders and human eyes is a lens that is characterized by transparency and a relatively soft consistency, as well as the absence of pigment in jumping spiders and the presence of pale yellow pigments in humans [13,15,60,68,78,79]. Based on photographs of sections of the eyes of jumping spiders, such as *M. aeneolus* [1], *Phidippus johnsoni* Peckham and Peckham, 1883 [1], *M. harfordi* [40], *T. planiceps* [33] and *P. magnifica* [38], in the lens of the antero-median, antero-lateral and postero-lateral eyes, it is possible to distinguish the anterior and posterior parts, which differ from each other in their size, size relative to the pupil, their shape and the shape of the refractive surface. In addition, the posterior part of the lens, depending on the species of jumping spider, for example, in *M. aeneolus* and *P. johnsoni* [1], may differ in the last two properties. In these eyes, in the given species the anterior smaller part of the lens is larger than the pupil and is crescent-shaped with a hemispherical shape of the refractive surface. The posterior large part of the lens, the size of which is comparable to the size of the pupil, in antero-median and antero-lateral eyes has a shape resembling the shape of an open truncated pyramid with a smaller base facing the eye cavity and with a flattened or hemispherical shape of the refractive surface; in postero-lateral eyes it has the shape of an open sphere with a hemispherical shape of the refractive surface or the shape of an open oblate or prolate ellipsoid with an aspherical shape of the refractive surface [1,33,38,40]. The lens of postero-median eyes is comparable in size to the size of the pupil and has the shape of an oblate ellipsoid with an aspherical shape of refractive surfaces, as in *M. aeneolus* [1] and *M. harfordi* [40]. In humans, the size of the lens exceeds the size of the pupil. The lens is capable of accommodation, during which its shape and size change, as well as the shape of the refractive surfaces and the radius of their curvature. At accommodation rest, the lens has the shape of an oblate ellipsoid with flattened anterior and convex posterior surfaces, the curvature of which decreases from the central zone to the peripheral, whereas at maximum accommodation stress, the lens becomes rounded, and both its surfaces—more convex and of equal curvature [27]. In jumping spiders, the lens has a secretory origin and a layered structure. It is formed from substances that are synthesized in the cells of the vitreous body, and then secreted into the eye cavity [40,66]. The lens is optically homogeneous and has a different refractive index in different eyes, which also differs between species of jumping spiders. In antero-median eyes, the refractive index of the lens is 1.37, as in *P. johnsoni*, or 1.41, as in *M. aeneolus*, in antero-lateral eyes—1.43, as in *P. johnsoni*, or 1.45, as in *M. aeneolus*, and in postero-lateral eyes—1.49, as in *P. johnsoni*, or 1.51, as in *M. aeneolus* [1]. In humans, the lens is formed by epithelial tissue. It distinguishes between a capsule, a single-layer single-row cubic epithelium on the inner anterior surface of the capsule and the lens substance of elongated hexagonal epithelial cells. The latter form layers of different densities with a variable refractive index—from 1.386 at the periphery to 1.406 in the center [15,27,79].

In jumping spiders and humans, the lens performs several identical functions: light-refracting, light-guiding, shaping, supporting and protective [13,15]. In jumping spiders, the lens of the postero-lateral eyes may reduce spherical aberration, the postero-medial eyes—reduce spherical aberration. The lens of all species of eyes, as well as the lens of other invertebrates and vertebrates, is most likely capable of absorbing ultraviolet radiation with wavelengths shorter than 300 nm, as well as infrared radiation [48]. In humans, the lens participates in accommodation, reduces spherical aberration, as well as chromatic aberration by attenuating radiation in the violet-blue part of the spectrum [27,68]. The lens completely absorbs ultraviolet radiation with wavelengths shorter than 300 nm and infrared radiation with wavelengths longer than 1900 nm [50].

Thus, the lens of jumping spiders and humans has more different properties and performs more of the same functions (Table 1).

## 7. The Vitreous Body of the Camera-like Eyes of Jumping Spiders and Humans

In the principle and secondary eyes of jumping spiders and human eyes, the vitreous body lies behind the lens and, in comparison with it, occupies a significant part of the eye cavity (Figure 1 and Figure 2) [1,27,40]. Both in jumping spiders and in humans, the vitreous body is the internal environment of the eyes, which is characterized by transparency and colorlessness [27,66]. In jumping spiders, the vitreous body is formed by epithelial tissue [66]. Judging by the photographs of eye sections, for example, *P. magnifica* and *S. americana*, in the principle eyes, epithelial cells are oriented differently and do not form structured layers [38]. It can be noticed that in light-loving species, such as *P. magnifica* [38], all epithelial cells have a rounded shape, whereas in shade-loving species, such as *S. americana* [38], they are more or less prismatic shape. These properties do not permit the attribution of the vitreous epithelium of the principle eyes to any particular type of epithelium. In the secondary eyes, the epithelial cells are arranged radially and form a single layer, as in *M. harfordi* [40] and *P. magnifica* [38]. In these light-loving and shade-loving species, all epithelial cells have a prismatic shape and nuclei in the basal part [38,40]. Therefore, the vitreous epithelium of the secondary eyes can be classified as a single-layer single-row prismatic epithelium. In the principle and secondary eyes, the epithelial cells are separated from the retina by the basement membrane [66]. In humans, the vitreous body is formed by loose fibrous irregular connective tissue, which consists of an extracellular matrix, represented by collagen fibers in the ground substance, and several types of cells, the main of which are hyalocytes. The vitreous body is surrounded by a hyaloid membrane that separates it from the retina, ciliary body, ligament of Zinn and lens [15,80]. In both jumping spiders and humans, the vitreous body has a refractive index of 1.336 [27,55].

In jumping spiders and humans, the vitreous body performs several identical functions: shaping, supporting, protective and light-conducting [15,66,72]. In jumping spiders, the vitreous body participates in the formation of the lens, completely absorbs ultraviolet radiation with wavelengths shorter than 300 nm and, along with other invertebrates and vertebrates, is most likely to absorb infrared radiation [22,48]. In humans, the vitreous body performs a metabolic function, and also completely absorbs ultraviolet radiation with wavelengths shorter than 300 nm and infrared radiation with wavelengths longer than 1400 nm [15,50,72].

Thus, the vitreous body of jumping spiders and humans has more of the same properties and performs more of the same functions (Table 1).

## 8. Conclusions

A comparative analysis of the principle and secondary camera-like eyes of jumping spiders and human camera-like eyes, carried out on the basis of literature data, made it possible to identify the similarities and differences in the properties and functions of their basic components. The outer shell of the eyes and the vitreous body have more of the same properties, while the inner shell of the eyes, the pupil and the lens—on the contrary—have more different properties. The outer and inner shells of the eyes, the lens and the vitreous body perform more of the same functions, while the pupil—on the contrary—more different functions. In general, of the five components considered of the principle and secondary camera-like eyes of jumping spiders and human camera-like eyes, the outer shell of the eyes, the lens and the vitreous body show more similarities, the inner shell of the eyes and the pupil—more differences.

## Figures and Tables

**Table 1:** Similarities and differences between the components of the principle and secondary camera-like eyes of jumping spiders and humans.

Properties and Functions of Components	Similarities (+) and Differences (−)
Outer Shell	
Cornea	
Properties	
Location in the eyes	+
Size relative to the size of the outer shell	+
Transparency	+
Pigmentation	+
Structure	−
Shape of refractive surfaces	−
Lens type	+
Refractive index	−
Functions	
Shaping	+
Supporting	+
Protective	+
Light-refracting	+
Light-guiding	+
Light filter for UV and IR radiation	+
Reducing spherical aberration	−
Eye Capsule/Sclera	
Properties	
Location in the eyes	+
Size relative to the size of the outer shell	+
Transparency	+
Pigmentation	+
Structure	−
Refractive index	+
Functions	
Shaping	+
Supporting	+
Protective	+
Muscle attachment site	+
Participation in the outflow of intraocular fluid	−
Inner Shell	
Retina	
Properties	
Mobility	−
Shape	−
Optic and non-optic part	+
Location of the optic part	−
The shape of the optic part	−
Location of the non-optic part	−
Fovea	+/−
The optic part consists of a fovea	−
Central and peripheral region in the optic part	+/−
Zones in the central and peripheral region of the optic part	−
Fovea shape	−
Fovea curvature	−
Fovea depth	−
Fovea width	−
The location of the fovea relative to the optical axis of the eye	−
Fovea pigmentation	−
The presence of all retinal layers in the fovea	−
Inversion	+/−
The type of tissue in the optic part	+
The number of layers in the optic part	?
Layers in the optic part	?
The number of cell types in the optic part	+/−
Types of cells in the optic part	+/−
The type of tissue in the non-optic part	+
The number of layers in the non-optic part	?
Layers in the non-optic part	?
The number of cell types in the non-optic part	+
Types of cells in the non-optic part	+
Extracellular matrix of the retina	?
Refractive index	+
Functions	
Shaping	+
Supporting	+
Light-guiding	+
Central vision	+/−
Color perception	+/−
Peripheral vision	+/−
Light perception	+/−
Perception of the depth of space based on monocular cues	+/−
Perception of the depth of space based on binocular cues	+/−
Reducing chromatic aberration	−
Pupil	
Properties	
Presence	+/−
Location relative to the cornea	+
Location relative to the lens	−
Location in the shell of the eye	−
Shape	−
Diameter change	−
Functions	
Penetration of light into the eye	+
Regulation of the amount of light entering the eye	−
Buffer when switching from bright light to dark	−
Influence on visual parameters through diameter change	−
A channel for aqueous humor between the anterior and posterior chambers of the eye	−
Lens	
Properties	
Location relative to the cornea	−
Size relative to eye size	−
Transparency	+
Consistency	+
Pigmentation	−
Size relative to pupil size	+/−
Shape	+/−
Shape of refractive surfaces	+/−
Structure	−
Optical homogeneity	−
Refractive index	+/−
Functions	
Light-refracting	+
Light-guiding	+
Shaping	+
Supporting	+
Protective	+
Participation in accommodation	−
Reducing spherical aberration	+/−
Reducing chromatic aberration	−
Light filter for UV and IR radiation	+
Vitreous body	
Properties	
Location in the eyes	+
Size relative to eye size	+
Transparency	+
Pigmentation	+
Structure	−
Refractive index	+
Functions	
Shaping	+
Supporting	+
Protective	+
Light-guiding	+
Participation in the formation of the lens	−
Light filter for UV and IR radiation	+
Metabolic	−

## Data Availability

The data presented in this study are available on request from the corresponding author.
